# Transoral plasma radiofrequency ablation for selected supraglottic and glottic laryngeal cancer: a propensity score–matched comparative study

**DOI:** 10.3389/fonc.2026.1915154

**Published:** 2026-07-23

**Authors:** Sanchun Wang, Jinqiu Li, Hongyan Wang, Baishuo Huang, Bo Teng

**Affiliations:** Department of Otolaryngology Head and Neck Surgery, The Second Hospital, Jilin University, Changchun, China

**Keywords:** glottic carcinoma, open surgery, supraglottic carcinoma, survival, transoral plasma radiofrequency ablation

## Abstract

**Introduction:**

Transoral endoscopic surgery is a preferred treatment for early-stage and selected advanced laryngeal cancers. Transoral plasma radiofrequency ablation (TPRA) has shown promise for glottic cancer, however, its efficacy in T1-T2 supraglottic and selected advanced T2-T3 glottic cancers requires further evaluation.

**Methods:**

We retrospectively compared 31 patients with T1-T2 supraglottic or T2-T3 glottic cancers treated with TPRA with 36 patients treated with open partial laryngeal surgery. Disease-specific survival (DSS), disease-free survival (DFS), and local control (LC) were analyzed using multivariable Cox proportional hazards regression. Postoperative outcomes and preservation of laryngeal function were also evaluated.

**Results:**

After propensity score matching, 67 patients were included in the analysis. The TPRA group had a shorter median hospital stay (8 days), and no patient required synchronous tracheotomy or experienced postoperative bleeding. Functional recovery was generally faster than in the open surgery group, including avoidance of routine nasogastric tube feeding. Temporary dysphagic cough (25.8%) and granulation tissue (16.1%) were observed. The 5-year DSS, DFS, and LC rates after TPRA were 77.4%, 71.0%, and 83.1%, respectively, which were comparable to those after open surgery (74.8%, 72.2%, and 84.9%, respectively; all P > 0.05).

**Conclusion:**

TPRA demonstrated oncologic efficacy comparable to open surgery for selected T1-T2 supraglottic and T2-T3 glottic cancers, with better functional preservation and fewer perioperative complications. Together with its technical adaptability, TPRA may represent a valuable treatment option for carefully selected patients.

## Introduction

For the surgical management of laryngeal cancer, the primary objective is not only oncologic control but also preservation of laryngeal function whenever feasible. Within this paradigm, transoral endoscopic surgery has become an important larynx-preserving approach. Compared with open partial laryngeal surgery, it is less invasive and may shorten hospitalization and reduce postoperative morbidity in appropriately selected patients.

TPRA operates in a saline medium and creates a controlled, relatively low-temperature plasma field. This allows tissue ablation and hemostasis to be performed simultaneously. It may also reduce collateral thermal injury to surrounding healthy mucosa and laryngeal structures. These features may help minimize intraoperative bleeding, reduce postoperative tissue stress, accelerate swallowing recovery, and shorten hospitalization. In selected patients, TPRA may also help preserve laryngeal function compared with open partial laryngectomy.

In addition, the device has a flexible distal tip. When used with angled endoscopes, this feature can improve maneuverability during transoral endoscopic surgery. These attributes support the use of TPRA as a potential transoral option for selected patients with laryngeal cancer. Previous studies have shown that TPRA can achieve oncologic outcomes comparable to open surgery for T1 glottic cancer in terms of survival and recurrence rates (1). However, evidence regarding its effectiveness in T1-T2 supraglottic and T2-T3 glottic cancers remains limited. Therefore, this retrospective study aimed to evaluate the therapeutic efficacy of TPRA in patients with T1-T2 supraglottic and T2-T3 glottic cancers.

## Methods

### Patients

We retrospectively analyzed 67 patients with T1-T2 supraglottic cancer or T2 glottic cancer, as well as a small number of patients with selected T3 glottic cancer, who were treated at the Department of Otolaryngology, The Second Hospital of Jilin University, between January 2015 and September 2022. Eligible tumors were limited in extent and showed no significant extension into adjacent structures, such as the paraglottic space, base of the tongue, postcricoid area, or inner surface of the thyroid cartilage, and no subglottic invasion greater than 5 mm. Involvement of the anterior commissure was not considered a contraindication. Among the 67 patients, 31 underwent TPRA and 36 underwent open surgery. Open surgery was selected when preoperative or intraoperative assessment indicated that TPRA could not be performed safely or adequately.

This retrospective study was approved by the Ethics Committee of The Second Hospital of Jilin University (approval No. 2025223). Tumors were classified according to the eighth edition of the American Joint Committee on Cancer (AJCC) TNM classification (2). No patient received adjuvant treatment before surgery.

Laryngoscopy, magnetic resonance imaging, computed tomography, ultrasonography, and positron emission tomography/computed tomography (PET/CT) when necessary were performed to assess tumor extent and metastatic status. All cases were histologically confirmed as laryngeal cancer by preoperative biopsy. Patients with distant metastasis or other malignant diseases were excluded.

### Surgical technique

**Figure 1:**
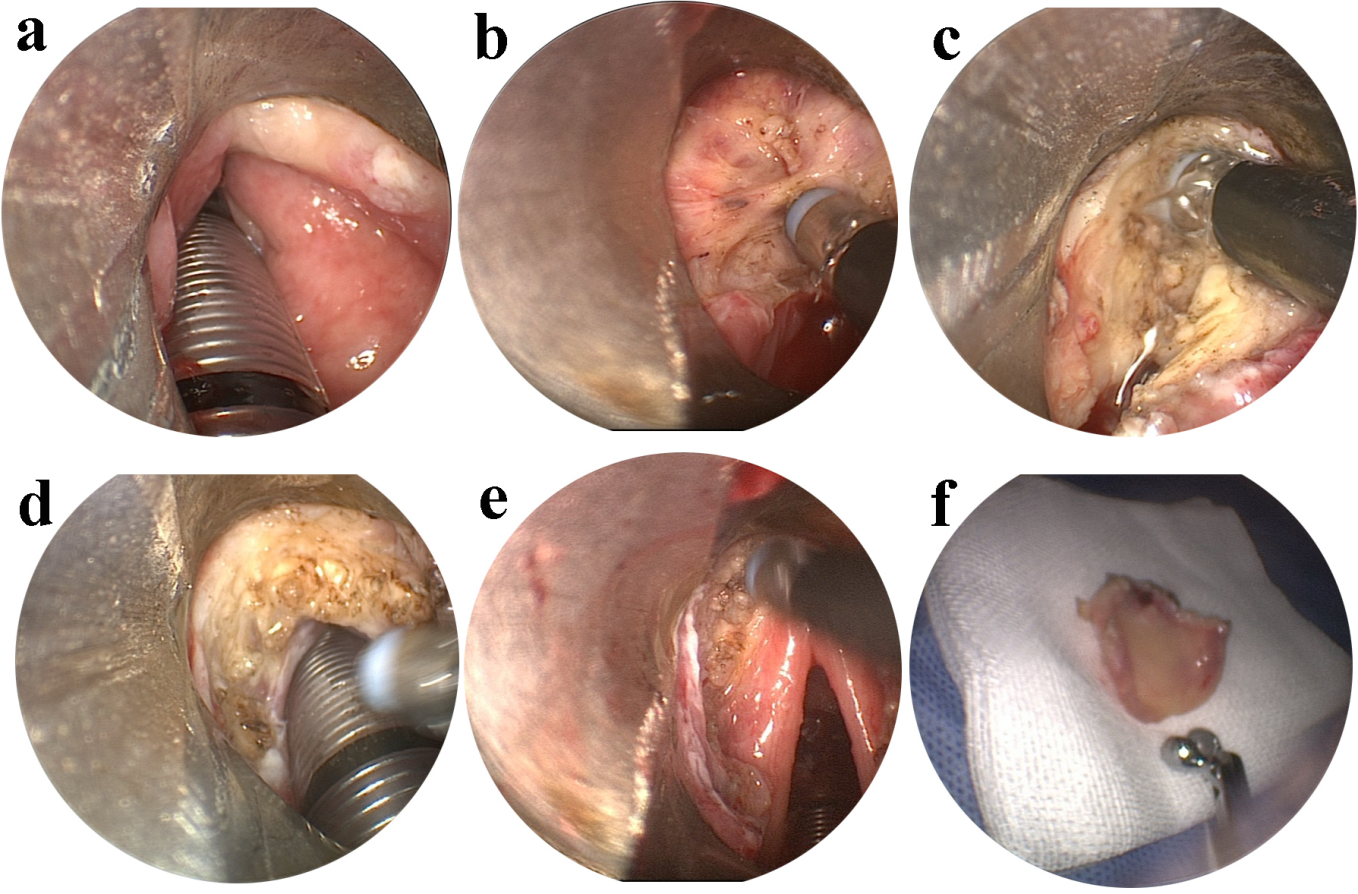
Representative TPRA procedure for supraglottic laryngeal cancer. **(a)** Preoperative endoscopic view showing the tumor location. **(b)** Intraoperative exposure and demarcation of the planned resection margin. **(c)** Tumor resection using TPRA. **(d)** Intraoperative view after tumor removal. **(e)** Reinspection of the resection bed and additional hemostasis. **(f)** Resected specimen.

After the tumor location and extent were determined, a resection line was marked approximately 5 mm from the tumor margin. The tumor was resected along this line, with en bloc removal attempted whenever feasible, and bleeding was controlled simultaneously to maintain a clear operative field. Depending on tumor location and extent, resection could involve the hyoid or thyroid cartilage. If cartilage invasion was suspected, one arytenoid cartilage could be removed. The bilateral superior laryngeal arteries could be ligated, and electrocoagulation was used for hemostasis. When exposure was partially limited, such as at the anterior commissure, an angled endoscope and a bent device tip were used to facilitate complete resection. The mean operative time was 40 minutes. If exposure was severely restricted, the procedure was converted to open surgery (**Figure 1**).

### Neck treatment

Patients with T3 glottic cancer underwent synchronous preventive selective neck dissection (ND). For patients with T2 glottic and T1-T2 supraglottic cancer, ND was performed only when imaging findings suggested possible cervical lymph-node metastasis. No synchronous tracheotomy was performed in the TPRA group.

### Postoperative treatment

Patients who underwent glottic surgery were allowed oral feeding after recovery from anesthesia, whereas those who underwent supraglottic surgery began oral feeding on postoperative day 2. Postoperative radiotherapy (RT) was administered four weeks after surgery to all patients with T2 supraglottic and T3 cancers. Other patients received RT only when adverse pathological features were present, including extranodal extension, positive or close margins, pT4 disease, pN1-N3 nodal involvement, perineural invasion, vascular invasion, lymphatic invasion, poor differentiation, or subglottic extension. Treatment planning was based on CT simulation, and postoperative RT was delivered using intensity-modulated radiotherapy (IMRT). The planning target volume (PTV) was defined according to tumor subsite, postoperative pathological risk factors, nodal status, and suspected subclinical spread. For high-risk patients, defined as those with adverse pathological features, the PTV dose was 60–66 Gy at 2.0 Gy per fraction. For low- to intermediate-risk patients with suspected subclinical spread, the PTV dose was 44–50 Gy at 2.0 Gy per fraction or 54–63 Gy at 1.6-1.8 Gy per fraction.

### Statistical methods

Multivariable Cox proportional hazards regression was used for multivariable analysis. Survival curves were generated using the Kaplan-Meier method. Disease-specific survival (DSS), disease-free survival (DFS), and local control (LC) rates were calculated from surgery to tumor-related death, recurrence, metastasis, or the last follow-up, as appropriate. A p-value of <0.05 was considered statistically significant.

## Results

To minimize potential baseline imbalances between the two groups, propensity score matching (PSM) was performed. After matching, 67 patients were included in the analysis, comprising 8 exactly matched pairs and 23 pairs generated by fuzzy matching. The propensity score distributions did not differ significantly between the two groups (P = 0.542).

Most patients were diagnosed with squamous cell carcinoma (SCC). In the TPRA group, one patient had neuroendocrine carcinoma with concomitant basaloid squamous cell carcinoma features. **Table 1** summarizes the baseline characteristics and treatment modalities of both groups. No significant differences were observed between the open surgery and TPRA groups regarding patient factors (age, gender), tumor characteristics (tumor location, T-staging, N-staging, differentiation grade, subglottic extension), or the use of RT and chemotherapy. However, the rate of ND differed significantly between the two groups (P < 0.001), which is attributable to the differing indications and approaches for neck dissection associated with each surgical method.

**Table 1:** Clinical characteristics of study participants.

Variables		Total (N = 67)	Open surgery (N = 36)	TPRA (N = 31)	P-value
Patient Factors	Age	62.81 ± 7.74	62.53 ± 7.24	63.13 ± 8.39	0.754
	Gender, n (%)				0.569
	Male	52 (77.6)	29 (80.6)	23 (74.2)	
	Female	15 (22.4)	7 (19.4)	8 (25.8)	
Tumor Characteristics	Tumor Location, n (%)				0.999
	Supraglottic cancer	28 (41.8)	15 (41.7)	13 (41.9)	
	Glottic cancer	39 (58.2)	21 (58.3)	18 (58.1)	
	T-staging, n (%)				0.999
	T1	6 (9.0)	3 (8.3)	3 (9.7)	
	T2	58 (86.6)	31 (86.1)	27 (87.1)	
	T3	3 (4.5)	2 (5.6)	1 (3.2)	
	N-staging, n (%)				0.678
	N0	60 (89.6)	31 (86.1)	29 (93.5)	
	N1	5 (7.5)	4 (11.1)	1 (3.2)	
	N2	2 (3.0)	1 (2.8)	1 (3.2)	
	Differentiation grade, n (%)				0.544
	Well	24 (35.8)	11 (30.6)	13 (41.9)	
	Moderately	39 (58.2)	23 (63.9)	16 (51.6)	
	Poorly	3 (4.5)	2 (5.6)	1 (3.2)	
	Other*	1 (1.5)	0 (0.0)	1 (3.2)	
	Subglottic extension, n (%)				0.999
	Yes	12 (17.9)	6 (16.7)	6 (19.4)	
	No	55 (82.1)	30 (83.3)	25 (80.6)	
Treatment Modalities	Neck Dissection, n (%)				<0.001
	Yes	31 (46.3)	27 (75.0)	4 (12.9)	
	No	36 (53.7)	9 (25.0)	27 (87.1)	
	Radiotherapy, n (%)				0.999
	Yes	31 (46.3)	17 (47.2)	14 (45.2)	
	No	36 (53.7)	19 (52.8)	17 (54.8)	
	Chemotherapy, n (%)				0.761
	Yes	12 (17.9)	7 (19.4)	5 (16.1)	
	No	55 (82.1)	29 (80.6)	26 (83.9)	

P-values represent comparisons between the Open Surgery and TPRA groups.

Continuous variables (e.g., age) are presented as mean ± standard deviation (SD) and analyzed using an unpaired t-test.

Categorical variables are presented as number (percentage) and analyzed using Chi-square test or Fisher’s exact test as appropriate.

*Other in “Differentiation grade” refers to one case of neuroendocrine carcinoma with concomitant basaloid squamous cell carcinoma features in the TPRA group.

**Figure 2:**
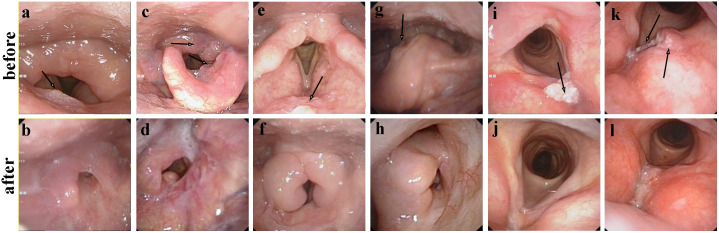
Preoperative and postoperative laryngoscopic images after TPRA surgery. Panels **(a, c, e, g, i, k)** show preoperative images, and panels **(b, d, f, h, j, l)** show the corresponding postoperative images. The arrows indicate the lesion locations.

Postoperative recovery in the TPRA group was generally favorable. The median hospital stay was 8 days (range, 5–13 days), and no patient experienced postoperative bleeding at the surgical site. As shown in **Figure 2**, surgical wounds after TPRA healed well across different anatomical locations.

Further analysis of the TPRA group showed that six patients (19.4%) had subglottic extension, all originating from glottic cancer. Positive surgical margins were identified in three patients (9.7%). Regarding regional lymph-node management, four patients (12.9%) underwent simultaneous unilateral or bilateral ND, and pathological neck metastasis was confirmed in two of these patients. Fourteen patients (45.2%) received postoperative adjuvant RT. The indications included T1 supraglottic cancer with N2 disease in one patient, T2 supraglottic cancer in five patients, and glottic cancer in eight patients, including five with subglottic extension, one with positive margins, and two with close margins.

In the open surgery group, six patients (16.7%) had subglottic extension, all originating from glottic cancer, and four patients (11.1%) had positive surgical margins. Twenty-seven patients (75.0%) underwent simultaneous ND, and pathological neck metastasis was confirmed in five patients. Postoperative adjuvant RT was administered to 17 patients (47.2%). The indications for adjuvant RT included T1 supraglottic cancer with close margins in one patient, T2 supraglottic cancer in nine patients, including cases with N1 disease, N2 disease, positive margins, or poor differentiation, and glottic cancer in seven patients, including cases with subglottic extension, positive margins, N1 disease, or T3 disease.

Seven patients (19.4%) in the open surgery group and five patients (16.1%) in the TPRA group received chemotherapy, all of which was initiated after recurrence or metastasis was detected.

### Oncologic results

In the TPRA group, follow-up ranged from 11 to 109 months, with a median of 65 months. Five patients (16.1%) developed local recurrence within 13–22 months, and four patients (12.9%) developed neck metastasis within 6–20 months after surgery. Seven patients (22.6%) died of tumor progression. In the open surgery group, follow-up ranged from 12 to 118 months, with a median of 61 months. Five patients (13.9%) developed local recurrence within 12–34 months. At the final follow-up, nine patients (25.0%) had died of tumor progression.

**Figure 3:**
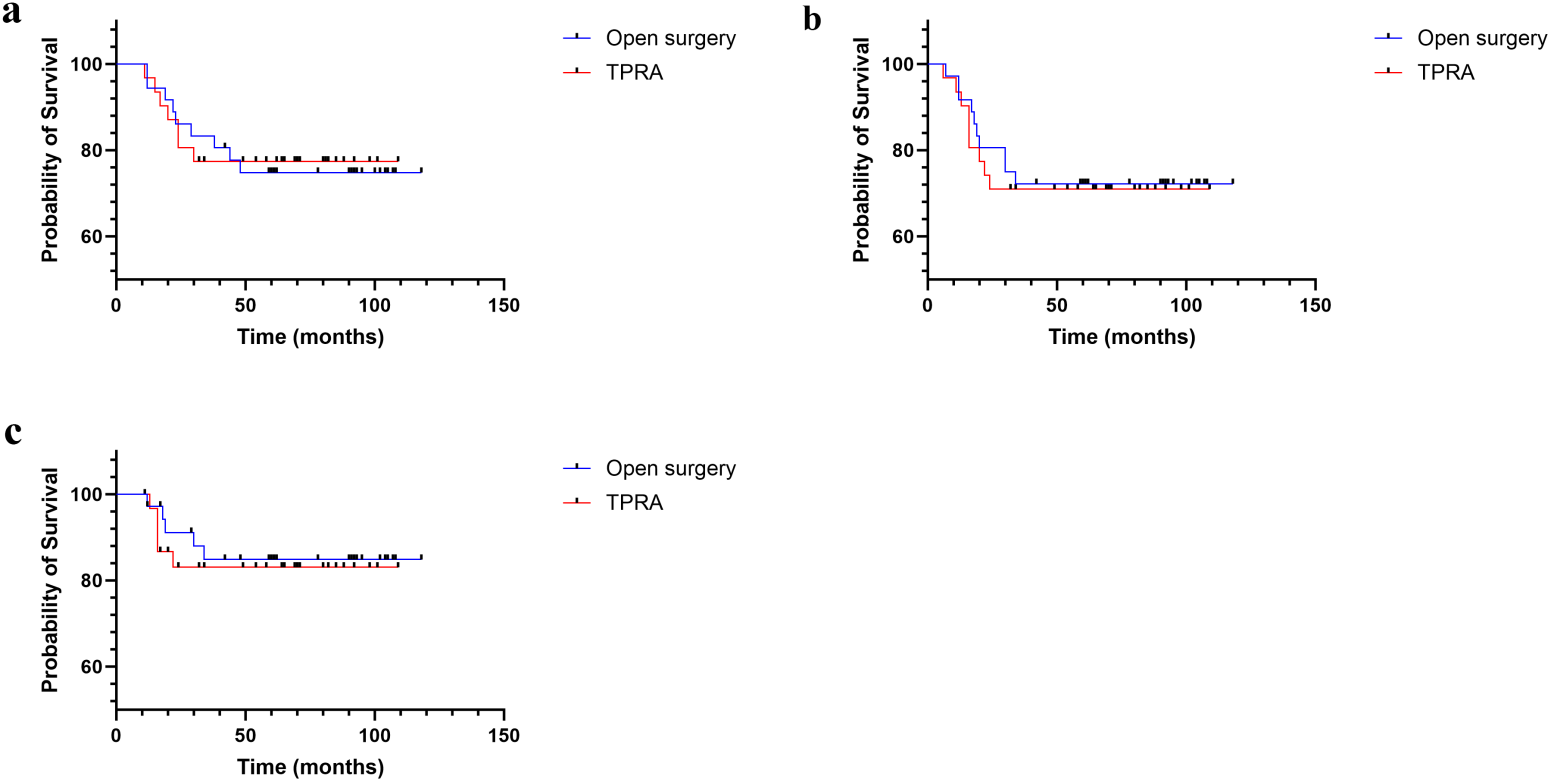
Comparison of DSS **(a)**, DFS **(b)**, and LC **(c)** between patients who underwent open surgery and TPRA for supraglottic and glottic cancer.

We further analyzed factors associated with survival using Cox proportional hazards regression. Compared with open surgery, TPRA was not associated with a significantly different risk of events for DSS (hazard ratio [HR] = 0.6306; 95% confidence interval [CI], 0.1364-2.649), DFS (HR = 1.368; 95% CI, 0.3579-5.469), or LC (HR = 2.166; 95% CI, 0.2912-19.07). The 5-year DSS, DFS, and LC rates were 74.8%, 72.2%, and 84.9%, respectively, in the open surgery group and 77.4%, 71.0%, and 83.1%, respectively, in the TPRA group. Kaplan-Meier analysis showed no significant differences in these outcomes between the two surgical approaches (all P > 0.05; **Figure 3**).

### Functional results

In the TPRA group, no patient required nasogastric tube feeding after surgery. Eight patients (25.8%) who underwent epiglottectomy or partial epiglottic resection experienced coughing or aspiration symptoms while eating or drinking during the first few postoperative days, these symptoms improved within 4–20 days. Three patients (9.7%) required temporary tracheotomy 1–16 months after surgery because of laryngeal edema, two cases of which were related to RT. Three patients (9.7%) underwent salvage surgery for local recurrence, including two with glottic cancer and one with supraglottic cancer. Five patients (16.1%) developed granulation tissue at the surgical site, three improved with conservative treatment, whereas two required resection of granulation tissue. One patient (3.2%) with T2 glottic cancer developed thyroid cartilage necrosis one month after surgery and underwent secondary surgery to remove the necrotic tissue. Systematic voice-function assessments were not available for all patients during follow-up.

In the open surgery group, all patients received postoperative nasogastric tube feeding until coughing or aspiration symptoms improved. The duration of tube feeding ranged from 11 to 90 days, with a median of 15 days. All patients underwent synchronous tracheostomy. Twenty-eight patients (77.8%) were decannulated between 2 weeks and 6 months after surgery, whereas eight patients (22.2%) still had a tracheostomy cannula at the last follow-up. Three patients (8.3%) with supraglottic cancer underwent total laryngectomy because of local recurrence. Eleven patients (30.6%) developed granulation tissue in the surgical area, but none required surgical resection of the granulation tissue.

## Discussion

For T1-T2 and selected T3 glottic and supraglottic laryngeal cancers, primary treatment modalities include transoral endoscopic surgery, RT, and open surgery. Open surgery can compromise the laryngeal framework and negatively affect postoperative quality of life. In contrast, endoscopic resection offers the potential for better laryngeal preservation while avoiding some RT-related complications. Our findings suggest that, in selected patients with T1-T2 supraglottic and T2-T3 glottic cancers, TPRA achieved DSS, DFS, and LC outcomes comparable to those of open surgery, while providing better functional preservation and fewer postoperative complications.

The role of synchronous ND during transoral laryngectomy remains debated. Some suggest a wait-and-see approach for early supraglottic cancer to reduce morbidity without negatively impacting survival (3), while others advocate for simultaneous ND (4). In the present cohort (TPRA), patients with T3 glottic cancer underwent synchronous preventive selective ND, whereas patients with T2 glottic and T1-T2 supraglottic cancer underwent ND only when imaging suggested possible cervical lymph-node metastasis. This approach may avoid unnecessary neck morbidity in patients without radiological evidence of nodal disease while still treating patients with more advanced glottic tumors or suspected nodal involvement. Ketterer et al. showed that pretherapeutic clinical nodal staging may overstage nodal disease, reported occult metastasis in only a minority of cN0 patients undergoing elective ND, and concluded that the indication for elective ND should be considered carefully, particularly in glottic laryngeal cancer. These findings support a cautious and selective approach to elective ND rather than routine ND for every cN0 patient (5). At the same time, the real world multicenter data reported by Knopf et al. emphasize that nodal positivity and tumor stage are important determinants of treatment outcome, and that supraglottic and hypopharyngeal tumors require particular caution because recurrence and salvage outcomes may be less favorable than in glottic cancer. Therefore, our imaging based neck management strategy should be interpreted in the context of careful preoperative staging, strict postoperative surveillance, and appropriate adjuvant RT when indicated. In this study, the different ND rates between TPRA and open surgery did not translate into a significant difference in DSS, DFS, or LC, however, the retrospective design and limited sample size preclude definitive conclusions regarding the optimal management of the clinically negative neck (6).

TPRA preserved thyroid cartilage integrity and facilitated rapid recovery of swallowing and speech. None of the 31 patients in the TPRA group required synchronous tracheotomy. However, glottic involvement may lead to adhesions that affect voice or breathing and may require tracheotomy or adhesiolysis. Long-term complications mainly included granulation tissue at the surgical site, which occasionally required secondary removal. Thyroid cartilage necrosis was rare and may have been related to device irritation or thermal effects after glottic tumor resection. In addition, because of the width of the cutting head, TPRA may cause some ablation effect at the resection edge, potentially contributing to excessive tissue damage or pathological findings such as positive or close margins.

Transoral laser microsurgery (TLM) is an established transoral surgical modality for early and selected advanced laryngeal cancers and provides an important benchmark for interpreting the clinical role of TPRA. Previous TLM studies have reported favorable oncologic outcomes for T1-T2 glottic and supraglottic cancers, as well as for selected advanced laryngeal cancers (7–13). For early supraglottic cancer, reported 5-year LC, DFS, and recurrence-free rates are approximately 76%, 69%, and 68.0%, respectively (8, 14), while a 5-year DSS of 86.7% has been reported for T2 glottic cancer (15). More recently, a large German multicenter cohort of T1/2 N0 M0 glottic cancer showed that surgical treatment achieved longer laryngeal preservation time than radiotherapy, further supporting the role of surgical larynx preservation strategies in early glottic cancer (16). In the present cohort, TPRA achieved 5-year LC and DSS rates that were broadly comparable to those reported in contemporary transoral surgical series. Notably, one patient with T3 glottic cancer treated with ipsilateral hemilaryngectomy in our cohort remained recurrence-free after 71 months of follow-up, suggesting that TPRA may be a promising approach for carefully selected advanced laryngeal cancers. However, because evidence for transoral surgery in advanced laryngeal cancer remains limited, careful patient selection is still required (17, 18). Regarding perioperative safety, our TPRA cohort showed a favorable profile. In a prospective multicenter TLM study, postoperative bleeding was reported in 3.9% of patients and laryngeal edema requiring tracheostomy in 1.0% (19). In our cohort, no postoperative bleeding occurred, one patient required tracheostomy for edema unrelated to immediate surgery, and all short-term aspiration symptoms resolved rapidly.

From a technical perspective, TPRA may provide complementary advantages in selected cases, particularly when exposure is restricted or intraoperative hemostasis is challenging. The flexible distal end of the device, combined with angled endoscopy, can facilitate resection in sites such as the anterior commissure. In addition, its simultaneous ablation and hemostatic effects may help maintain a clear operative field, reduce intraoperative bleeding, and avoid the risk of airway burns. Nevertheless, because the present study compared TPRA with open laryngeal surgery rather than with TLM, these findings should not be interpreted as demonstrating superiority or non-inferiority of TPRA compared with TLM. Direct comparative studies are needed to clarify the relative indications, functional outcomes, complication profiles, and long-term oncologic results of TPRA and TLM.

This study has several limitations. First, the sample size of the TPRA group was limited, and larger multicenter studies are needed to draw more robust conclusions. Second, postoperative laryngeal function was not evaluated using a uniform prospective protocol, and patient compliance was suboptimal, resulting in incomplete voice and swallowing assessments in some cases. Third, this retrospective study did not include a direct TLM control group, which limits comparison with the most widely established transoral surgical approach for early-stage laryngeal cancer. Fourth, neck dissection indications differed according to tumor stage, subsite, and imaging findings: patients with T3 glottic cancer underwent synchronous preventive selective neck dissection, whereas those with T2 glottic and T1-T2 supraglottic cancer underwent neck dissection only when imaging suggested possible nodal metastasis. This institutional strategy may limit comparison with centers that routinely perform elective neck treatment for selected cN0 subsites or stages, and it should be considered when interpreting regional control outcomes.

## Conclusion

TPRA offers survival outcomes comparable to open surgery for selected T1-T2 supraglottic and T2-T3 glottic cancers while improving laryngeal function preservation, avoiding routine synchronous tracheotomy, and reducing postoperative complications. Its excellent hemostatic control minimizes postoperative bleeding and avoids the risk of airway burns. Moreover, the flexible distal end of the device facilitates tumor resection in areas with partially limited visibility. Collectively, these advantages support TPRA as a valuable treatment option for carefully selected supraglottic and glottic cancers.

## Data Availability

The datasets analyzed during the current study are not publicly available because of patient privacy and ethical restrictions, but are available from the corresponding author upon reasonable request.
